# Improved quadriceps efficiency with a medial pivot in comparison to a cruciate‐retaining design in total knee arthroplasty

**DOI:** 10.1002/ksa.12624

**Published:** 2025-02-13

**Authors:** Leandra Bauer, Thomas R. Niethammer, Christoph Thorwächter, Matthias Woiczinski, Peter E. Müller, Florian Simon, Boris M. Holzapfel, Johanna‐Maria Simon

**Affiliations:** ^1^ Department of Orthopaedics and Trauma Surgery Musculoskeletal University Center Munich (MUM), University Hospital Munich, LMU Munich Munich Germany; ^2^ Experimental Orthopaedics University Hospital Jena, Campus Eisenberg, Waldkliniken Eisenberg, Friedrich‐Schiller‐University Jena Jena Germany; ^3^ Department of Otorhinolaryngology LMU University Hospital, LMU Munich Munich Germany

**Keywords:** cruciate retaining, knee rig, medial pivot, medial stabilized, total knee arthroplasty

## Abstract

**Purpose:**

The posterior cruciate‐retaining (CR) design offers rotational freedom but risks abnormal kinematics and instability. The medial pivot (MP) design mimics native joint motion with a high‐conformity medial and flat lateral interface. Within clinical studies, the MP design outclassed the CR design, but biomechanical studies are lacking. This study investigates the tibiofemoral and patellofemoral kinematics of both implant designs compared to native kinematics.

**Methods:**

Eight fresh‐frozen cadaveric knee specimens underwent total knee arthroplasty using MP and CR designs. Testing was performed in a dynamic knee rig simulating active knee flexion (30–130°) under muscle load. Biomechanical assessments included tibial rotation, tibiofemoral translation, patellar tilt/shift, patellofemoral contact/pressure patterns and quadriceps force. Functional regressions were used to analyse the effects of the component designs on the native situation.

**Results:**

The MP design exhibited increased tibial rotation (130° flexion: MP 9.4° vs. CR 6.6°) and lateral anterior tibial translation during flexion (130° flexion: MP 25.8 mm vs. CR 22.6 mm). Both designs showed no significant differences in patellar tilt or shift and similar patellofemoral pressure (CR 3.2 MPa, MP 3.4 MPa) and contact patterns (CR 213.8 mm^2^ vs. MP 230.4 mm^2^). The MP design required lower quadriceps force, particularly in deep flexion (NS 452.6 N, CR 407.8 N and MP 367.3 N).

**Conclusion:**

The MP design provides a more native‐like knee kinematic profile than the CR design, with a more pronounced MP motion pattern and reduced quadriceps loading.

**Level of Evidence:**

Not applicable.

**Abbreviations**
AP: anterior‐posterior
CR: cruciate retaining
GRF: ground reaction force
KA: kinematic alignment
MP: medial pivot
NS: native situation/state
RCT: randomized clinical trial
TKA: total knee arthroplasty

## INTRODUCTION

A wide range of implant designs have been developed to restore native joint kinematics after total knee arthroplasty (TKA) [8].

The posterior cruciate (ligament) retaining (CR) design features low conformity of the insert, potentially enabling rotational freedom and high amounts of femoral rollback on the medial and lateral sides [22]. However, several studies demonstrated abnormal kinematic patterns within CR TKA designs [3]. The lower contact area of the CR design, resulting in a reduction of anterior‐posterior (AP) stability, may also contribute to higher rates of mid‐flexion instability [35].

Based on findings regarding native knee joint kinematics, the medial pivot (MP) design was developed in the early 2000s [4, 17]. The MP TKA design presents a flat insert laterally to enable lateral femoral rollback and high conformity medially interacting as a so‐called ‘ball‐in‐socket’ joint, which is thought to replicate physiological kinematics and reduce contact stress [20].

Within a group of patients with bilateral TKA, 76% identified the MP TKA as the ‘overall better knee’ compared to their opposite CR TKA [25]. Furthermore, MP TKA led to better Forgotten Joint and KOOS scores, as well as better postoperative knee flexion and midrange AP knee stability [10, 12, 32]. However, registry data from Australia and Norway from 2005 to 2017 revealed a decreased Kaplan−Meier survival for MP TKA compared to CR TKA, depending on the specific implant manufacturer used [21].

Using fluoroscopic analyses, an MP motion similar to native joint kinematics could be verified for MP TKA, whereas the CR design generated a paradoxical roll‐forward motion of the femur relative to the tibia [26]. However, biplanar in vivo images may simply approximate physiological movements.

However, except for a finite element method analysis by Shu et al., in vitro analyses of tibiofemoral and patellofemoral kinematics and joint pressure loads comparing MP and CR TKA designs are lacking [27]. However, biomechanical data on knee joint kinematics is needed for evaluation and further development of implant design to reduce the rate of persistent complaints after TKA.

Therefore, the purpose of this study was to examine the influence of MP TKA compared to CR TKA on tibiofemoral and patellofemoral joint biomechanics on the baseline of native knee joint kinematics. Hereby, we hypothesized that an MP TKA would reproduce tibiofemoral and patellofemoral kinematics closer to native kinematics than a CR TKA.

## MATERIALS AND METHODS

### Preparation of the specimen

Ethical approval for this study was granted by the Ethics Committee of the University of Munich (#20‐829). Eight fresh‐frozen human cadaveric specimens (four males and four females) with an average age of 80 (±6) years were used for biomechanical testing. All specimens had a neutral hip–knee–ankle (0 ± 3°) and distal apex joint line obliquity of varying extent corresponding to CPAK type II [19], assessed via x‐ray fluoroscopy.

Skin and fat tissues of the specimen were removed, the muscles were dissected and the tendons of the quadriceps femoris, biceps femoris and semitendinosus muscles were secured in finger clamps using a FibreWire suture (Arthrex). The tibia and femur were shortened to 22 and 20 cm, respectively, as measured from the anatomical epicondylar axis. To ensure stability during the setup, the fibular head was fixed to the tibia using a cortical screw. Each knee joint was secured in the knee rig by embedding the tibial and femoral ends in metal pots, filled with epoxy resin, which can be directly mounted onto the dynamic knee rig [13, 29]. To avoid malalignment of the femur within the metal pots, the femoral posterior condyles were aligned parallel to the hip flexion axis of the experimental setup in the transverse plane.

### Prostheses and TKA implantation

**Figure 1:**
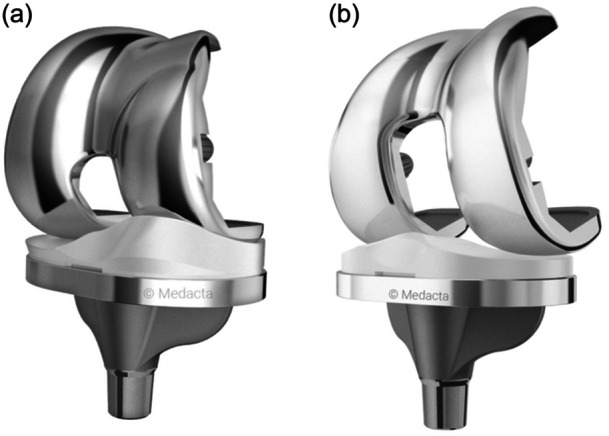
(a) MP TKA (Medacta GMK Sphere) (with permission of Medacta International, Castel San Pietro, Switzerland). (b) CR TKA (Medacta GMK Primary) (with permission of Medacta International, Castel San Pietro, Switzerland). CR, cruciate retaining; MP, medial pivot; TKA, total knee arthroplasty.

For TKA, an MP design (Figure 1a; GMK Sphere, Medacta International) and a CR design (Figure 1b; GMK Primary, Medacta International) were used. Both implant designs are used with the same tibial base plate, which was implanted once and had not been exchanged during the testing period. To ensure comparability and enable biomechanical testing, the femoral component and the insert had to be exchanged during the trial.

Kinematically aligned (KA) TKA has been performed, as KA TKA has been shown to maximize the primary concept of MP TKA in comparison to a mechanically aligned TKA [20]. TKA was performed by the senior author with regard to the technique of caliper‐verified unrestricted KA, introduced by Howell et al. and according to the manual by Weber and Gollwitzer [16, 36]. The posterior cruciate ligament was retained and the patella remained unresurfaced.

Due to inconsistent amounts of cartilage loss within the healthy specimen, a distal cut reference labelled ‘worn’ for medial and lateral condyles was used after resection of remaining cartilage, receiving distal femoral cuts of 6 mm thickness. Femoral component rotation was aligned to the posterior condyle axis. The tibial cut matched the coronal proximal tibial joint line and the individual medial tibial slope. The AP axis of the tibial baseplate was oriented parallel to the flexion‐extension plane as targeted in KA.

A 10 mm polyethylene insert was used in every TKA and x‐ray fluoroscopies as well as nano‐computed tomographies were performed before and after implantation to verify correct implant positioning. Switching from CR to MP design and therefore exchanging the femoral component was feasible without any difficulties, as both implant designs use the same 4‐in‐1 cutting block and peg holes.

### Biomechanical setup

The experiments were conducted using an established knee testing rig [28, 29]. This apparatus offers six degrees of freedom and is capable of performing an active knee flexion under load, ranging from 30° to 130°, with a constant ground reaction force (GRF) of 50 N. The GRF was maintained by employing a sensor‐controlled rectus femoris muscle (8417‐6002 Burster). To simulate muscle activity of the vastus medialis, vastus lateralis, semitendinosus, and biceps femoris, 2 kg weights were affixed to the respective tendons. Real‐time movement control was achieved through a custom‐developed LabView program (version 8.6, National Instruments). An optoelectrical measurement system (ARAMIS 3D Camera 2.3 M, GOM GmbH, resolution of 1936 × 1216 pixels, measuring volume of 1230 mm × 790 mm × 790 mm, accuracy 24.6 µm in focus‐plane and 49.2 µm out of focus‐plane) recorded the motion of the specimens by tracking markers positioned on the femoral and tibial heads, as well as on the patella. To assess retropatellar pressure distribution, a thin, pressure‐sensitive film (K‐Scan 4000, Tekscan Inc.) was placed on the retropatellar surface after opening the joint capsule [28]. First, the native situation (NS) was tested. Subsequently, measurements for CR TKA and MP TKA were performed.

### Data analysis and statistics

Data from the optoelectrical measurement system were synchronized and interpolated with the flexion angle recorded by the knee rig program. A well‐established method was employed for this analysis [2, 13, 29]. Subsequent data analysis was conducted using a custom MATLAB script (MathWorks Inc.). Peak pressure was calculated by averaging the maximum value over a window of eight surrounding values to avoid artefacts, according to Li et al. [18]. Contact area was determined by the number of pixels that exceeded a value of 0 MPa. AP translation was analysed as the global translation of the distal femur (i.e., translation of the midpoint of the condyles in relation to the tibia) and as a separate AP translation of the medial and lateral condyles. The femorotibial kinematics were described for a fixed femur, thereby demonstrating the movement of the tibia. The initial translation/rotation at 30° flexion was designated as the starting position for all kinematic analyses. The kinematic calculations were performed using the methodologies established by Bull et al. and Grood and Suntay [5, 15]. The flexion facet centres of the posterior condyles were projected onto the insert/tibial plateau and graphically connected to represent knee rotation. Measurements were taken at flexion increments of 5° or 10°, ranging from 30° to 120°. With the positions at 30° knee flexion serving as a reference, the straight lines were averaged across all specimens, with the standard deviation calculated in both the *x*‐ and *y*‐directions.

The results represent the average values across eight knee joints, with a 95% confidence interval (CI). Statistical analysis was performed using R Studio software (R version 4.3.1), where functional regressions (pffr), that is, function‐on‐scalar, were calculated utilizing the refund package (version 0.1‐35) [14]. The NS was used as a reference variable to assess the effects of CR and MP TKA on the target variables, which included quadriceps force, peak pressure, contact area and femorotibial and patellofemoral kinematics throughout the flexion cycle. Consequently, the results reflect the fixed impact of the NS in the intercept of the findings. The significance level was set at 0.05.

## RESULTS

### Femorotibial kinematics

**Figure 2:**
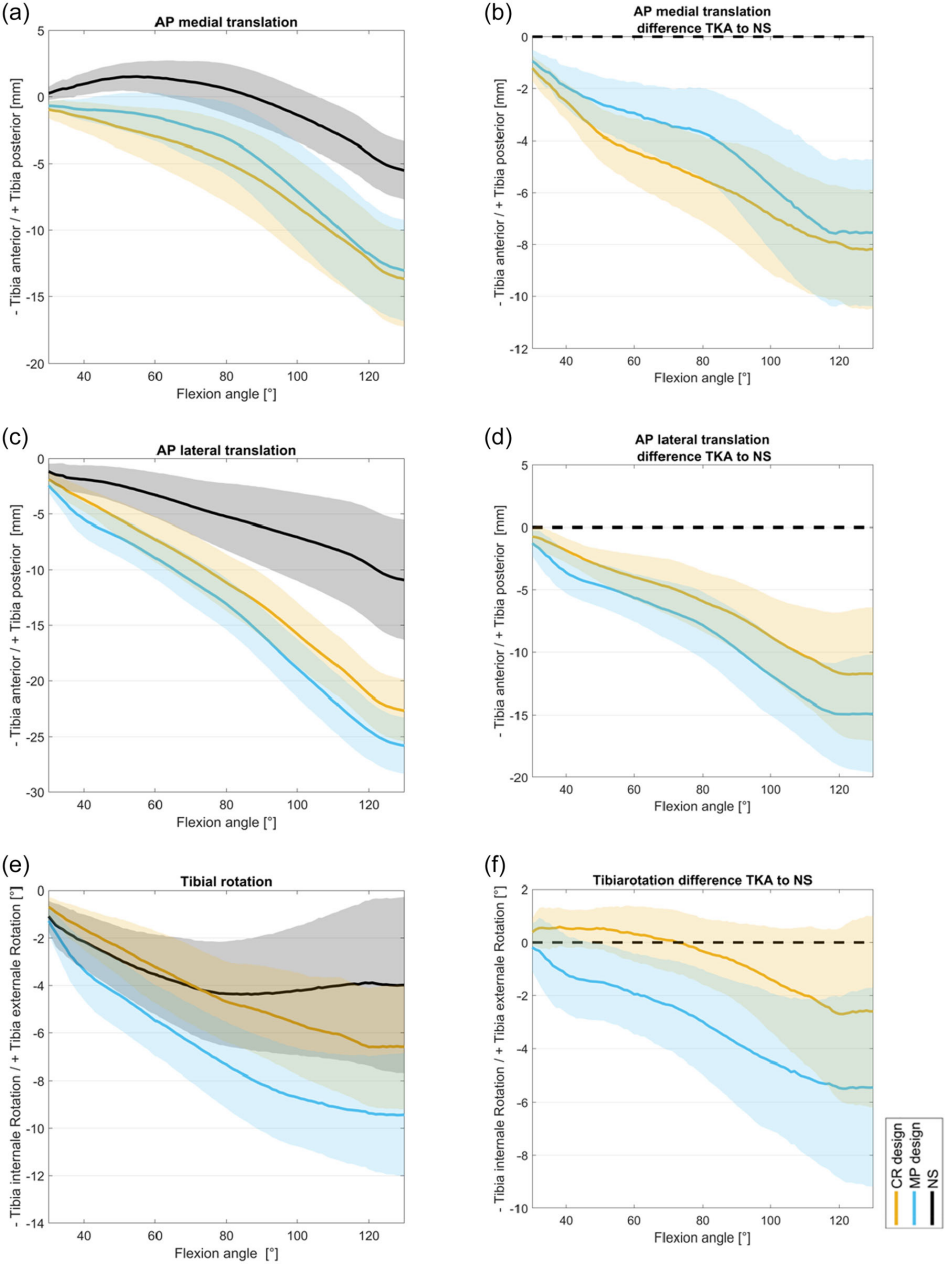
Femorotibial kinematics with (a) AP translation medial; (b) difference to NS for AP translation medial; (c) AP translation lateral; (d) difference to NS for AP translation medial; (e) tibial rotation and (f) difference to NS for tibial rotation; mean and 95% confidence interval for CR design (orange), MP design (blue) and native situation (black). AP, anterior‐posterior; CR, cruciate retaining; NS, native situation/state; TKA, total knee arthroplasty.

The analysis of the initial 70° of flexion demonstrated that just minimal anterior translation of the medial tibia occurred in the NS, suggesting the presence of a medial rotational point. This rotational point was better replicated by the MP design, that is, at 80° flexion 3.1 (±3.9) mm translation for MP TKA vs. 4.9 (±3.9) mm translation for CR TKA. In deeper flexion, all variants demonstrated anterior translation of the medial tibia (Figure 2a,b).

An anterior tibial translation laterally was more pronounced within MP TKA compared to CR TKA, that is, at 130° flexion, MP TKA 25.8 (±3.7) mm vs. CR TKA 22.6 (±4.1) mm. Functional regression analysis indicated that the lateral anterior tibial translation had a significantly greater impact on overall tibial AP movement than the medial side (Figure 2c,d).

Within functional regression analysis, the influence of a TKA (CR and MP) on AP kinematics generally increased with deeper flexion. Both implant designs showed significant effects (*p* < 0.001) on NS but did not differ from each other (see Supporting Information).

MP TKA exhibited greater internal tibial rotation throughout the entire flexion cycle (30–130°) with 9.4 (±3.7)° at 130° of flexion in comparison to CR with 6.6 (±3.8)° as seen in Figure 2e,f. Functional regression analysis indicated that MP TKA had a greater impact on tibial rotation than CR TKA, particularly in deep flexion. CR TKA exerted less influence up to 80° of flexion. Both the MP and CR TKA had significant effects (*p* < 0.001).

**Figure 3:**
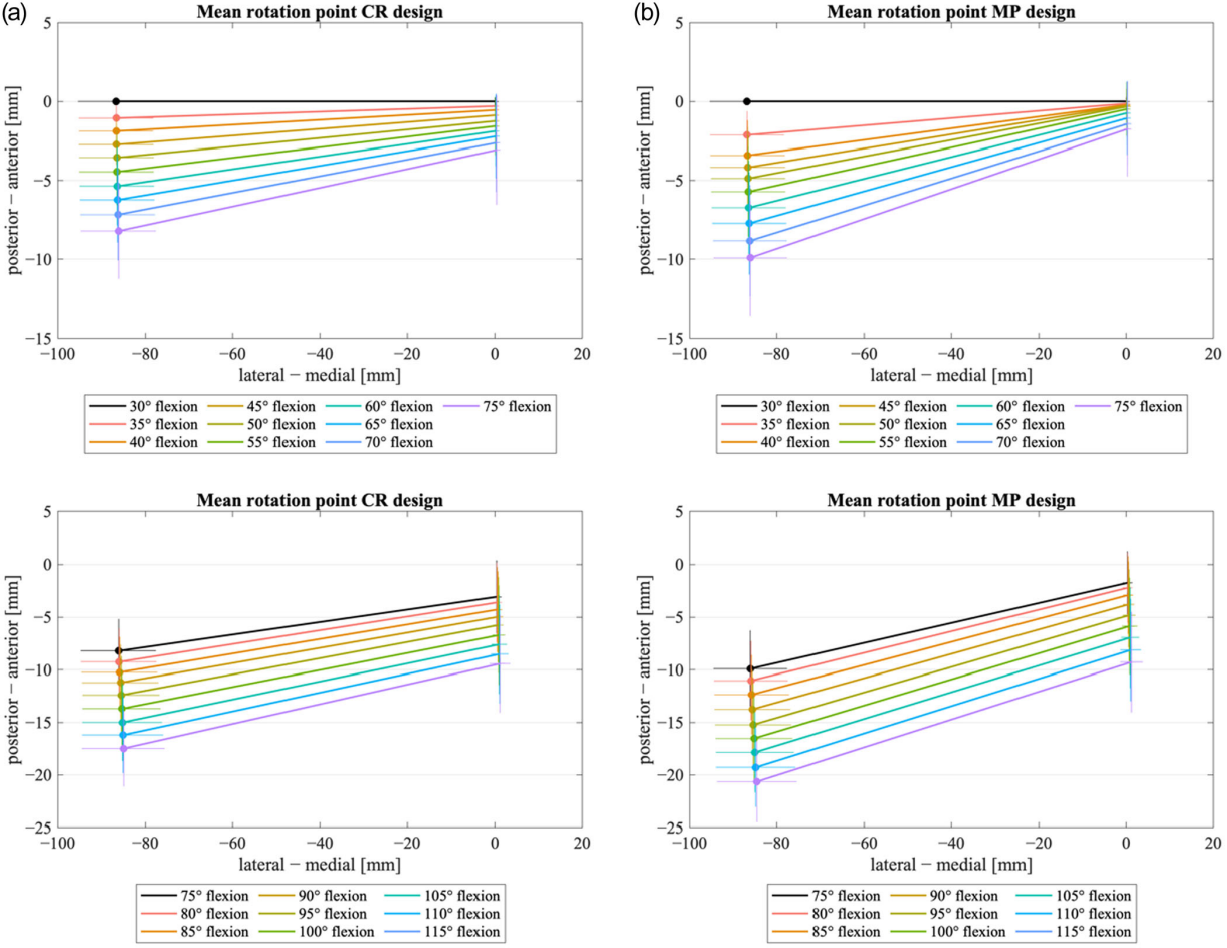
Mean rotation point of femur on tibia plateau for (a) CR design (35–75°; 75–115° flexion) and (b) MP design (35–75°; 75–115° flexion). CR, cruciate retaining; MP, medial pivot.

Figure 3 represents the projection of the femoral condyles onto the surface of the insert, showing the pivot point according to Pinskerova et al. [17]. For lower flexion angles (30–75°), a clearer rotation point around the medial femoral condyle for MP TKA became visible (posterior translation of the medial femoral condyle of 1.74 ± 3.01 mm for MP TKA vs. 3.07 ± 3.47 mm for CR TKA). The lateral femoral condyle translated posteriorly 12.92 ± 3.64 mm for MP TKA and 12.01 ± 3.03 mm for CR TKA within 30–75° of flexion. Within deeper flexion up to 120° the medial femoral condyle translated posteriorly 10.42 ± 4.71 mm for MP TKA and 10.39 ± 4.53 mm for CR TKA. The lateral femoral condyle translated posteriorly 24.92 ± 3.93 mm for MP TKA and 22.77 ± 3.76 mm for CR TKA. In the overall comparison, CR TKA showed a higher amount of posterior translation of the medial femoral condyle for lower flexion angles and simultaneously a reduced lateral femoral rollback within the whole flexion cycle.

### Patellofemoral kinematics

**Figure 4:**
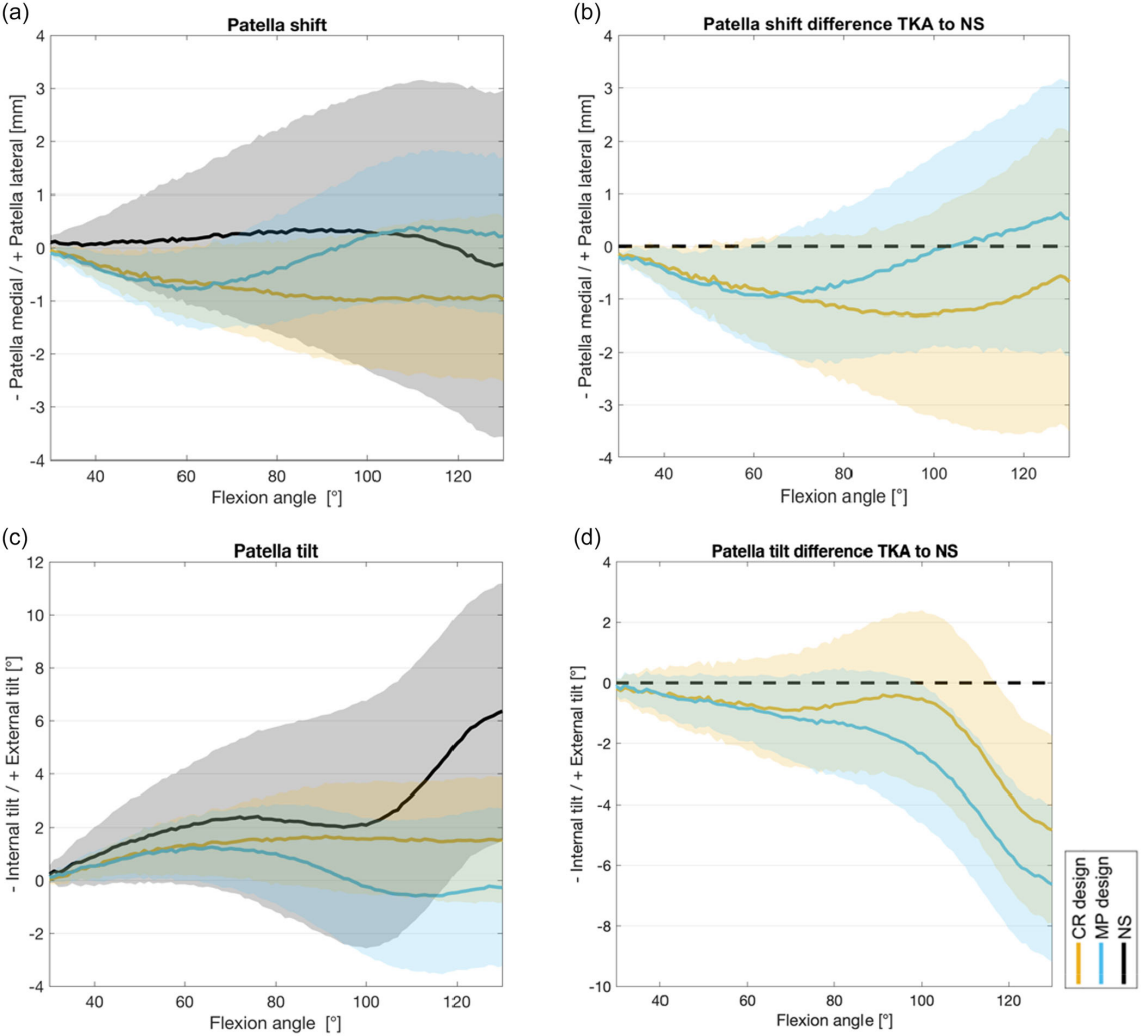
Patellofemoral kinematics for (a) patella shift, (b) difference to NS for patella shift, (c) patella tilt and (d) difference to NS for patella tilt; mean and 95% confidence interval for CR design (orange), MP design (blue) and NS (black). CR, cruciate retaining; MP, medial pivot; NS, native situation/state.

After TKA, the patellar shift demonstrated a medial movement in lower degrees of flexion. The patella within CR TKA remained in a medial position during deep flexion (1.0 mm medial shift), while MP TKA showed slight lateralization (from 0.8 mm medial to 0.4 mm lateral shift) of the patella back to the centre as knee flexion increased (Figure 4a,b). Regarding patellar tilt, there was minimal external tilt observed in moderate flexion up to 80°, with negligible differences between implant designs and the NS (ROM NS 2.4°, MP 1.3° and CR 1.6° tilt) (Figure 4c,d).

A high variance in native patellofemoral kinematics in between the specimens leads to overall high interindividual differences in patella shift and tilt, resulting in minor effects of the intercept within functional regression analysis (see Supporting Information).

### Knee joint loading

**Figure 5:**
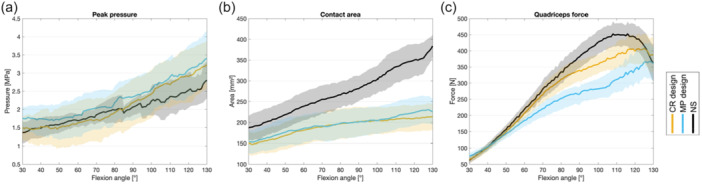
Knee joint loading represented by peak pressure (a), contact area (b) and quadriceps force (c) for CR design (orange), MP design (blue) and native situation (black) with mean and 95% confidence interval. CR, cruciate retaining; MP, medial pivot.

For patellofemoral peak pressures, significant differences between implant designs were observed in deeper flexion: max. values for NS 2.8 MPa, CR TKA 3.2 MPa and MP TKA 3.4 MPa (Figure 5a). However, these effects were minor, with differences of less than 1 MPa.

Both MP and CR TKA resulted in a significantly reduced patellofemoral contact area (*p* < 0.001) but without any relevant differences between the implant designs (Figure 5b), that is, for NS 384.0 mm^2^, CR TKA 213.8 mm^2^ and MP TKA 230.4 mm^2^.

Notable differences were depicted in quadriceps force between the implant designs. Overall, the MP design required less quadriceps force compared to the CR design (max. values NS 452.6 N, CR 407.8 N and MP 367.3 N) (Figure 5c). The MP design exhibited reduced quadriceps forces compared to NS and CR after 50° of flexion. In functional regression analysis, the CR design had almost no impact on quadriceps force compared to NS up to 80° of flexion. Afterwards, the impact increased until a flexion angle of 115°, then re‐decreased until full deep flexion. In contrast, the MP design exhibited an overall greater impact on changes in the quadriceps force of the NS, especially in deeper flexion degrees (see Supporting Information).

## DISCUSSION

The main findings of this study were an increased tibia rotation and anterior translation of the lateral tibia within the MP design compared to the CR design during flexion motion. Consequently, we could also observe a reduction of quadriceps load, assumingly due to the enlargement of the lever arm. Regarding patellofemoral kinematics (patella shift and tilt) and pressure loads, no relevant differences between MP and CR design were detected.

Our findings of tibiofemoral kinematics of the MP design contribute to the reconstruction of native kinematics according to Pinskerova et al., describing an MP motion pattern [24].

As the knee rig has a limited range of motion starting at 25–30° of flexion due to technical reasons, the study lacks contingency to compare the screw‐home mechanism of the knee. However, starting from 30° of flexion, a minor posterior translation of the medial femoral condyle within MP TKA in comparison to CR TKA (1.7 mm [MP] vs. 3.07 mm [CR]) until 75° of flexion and an equal amount of posterior translation exceeding 120° of flexion (10.42 mm [MP] vs. 10.39 mm [CR]) could be observed. The MP design also presented with a higher amount of posterior translation of the lateral femoral condyle (120° flexion: 24.92 mm for MP TKA vs. 22.77 mm for CR TKA) and a simultaneously increased internal tibia rotation (9.4° for MP TKA vs. 6.6° for CR TKA), better indicating the so‐called MP motion, as mentioned above. However, the absolute value of internal tibia rotation is reduced due to the inability to measure screw home motion.

Notably, our findings of native kinematics (NS) show less tibial rotation and anterior tibial translation within flexion compared to TKA which conforms to findings of earlier studies of our group, using the same knee rig and Varadarajan et al. using a similar knee rig. This might be caused by differences between the simulated and in vivo muscle forces [2, 11]. Yet, a weight‐bearing knee rig is considered a valuable instrument for the evaluation of key kinematic features of the native knee [34], especially as it has been shown that a mobile MP design may lead to different motion patterns in nonweight‐bearing conditions in comparison to knee motion during squatting [33].

A significant reduction of quadriceps load needed for active knee joint flexion could be observed for the MP design mainly at higher flexion rates (90–110°). This coincides with higher amounts of tibia rotation and anterior translation of the lateral tibia and hereby establishes an optimization of the quadriceps lever arm. This is maintained by the highly congruent implant design on the medial side within MP TKA, preventing anterior translation and shortening of the quadriceps lever arm [7]. In contrast, as a result of the flat surface design, CR TKA is often associated with paradoxical roll forward motion of the femur during flexion which on the one hand leads to a shortening of the quadriceps lever arm and also results in a sense of (mid‐flexion) instability [26, 27]. Those findings of an enlarged quadriceps lever arm when favoring the MP design for TKA could contribute to reduced knee muscle strain during activities of daily living. Even though the reduction of quadriceps load does not represent native kinematics, a reduced quadriceps load after TKA may be beneficial, as patients with osteoarthritis of the knee show an overall quadriceps weakness, assumingly due to arthrogenous muscle inhibition, which persists after TKA [31]. This assumption is supported by the clinical study of Pritchett et al., where 76% of patients with bilateral TKA favoured the MP over the contralateral CR‐designed TKA [25].

Although a reduction of quadriceps load may reduce quadriceps tension and patellofemoral compressive forces, within our study, we could not detect a reduction of patella peak pressure with either implant design used. However, it can be assumed that a reduction of contact area and an increased internal patella tilt after TKA compared to NS is mainly due to femoral component design regarding the trochlea (i.e., position and trochlear angle) and independent of the design of the femorotibial insert [30].

So far, two randomized controlled clinical studies (RCT) compared the outcome of CR TKA versus MP TKA designs via clinical outcome scores, each using varying implants and both KA and mechanical alignment techniques. The study by French et al. using KA revealed better Forgotten Joint Scores and Knee Injury and Osteoarthritis Outcome Scores within the MP TKA group but no difference in range of motion [12]. An RCT by Dowsey et al. compared MP TKA to CR TKA and a posterior‐stabilized TKA design [9]. Hereby, the MP TKA group showed superior outcomes in terms of pain, function and quality of life as well as Knee Society Scores. Hence, these clinical studies may support our findings of a more native reconstruction of knee kinematics when using an MP TKA design, assuming that a more ‘natural feeling of the knee’ contributes to a better clinical outcome.

Recent systematic reviews demonstrated high survivorship and low revision rates of MP TKA with comparable results to traditional TKA designs across several international joint registries [1, 6]. However, a current network meta‐analysis comparing clinical outcomes of posterior‐stabilized, CR and MP implant designs uncovered merely small and insignificant differences [23].

The presented study has a few limitations. First, as an in vitro study using a human cadaver specimen, the experimental setup performs a loaded squat via muscle simulation, but cannot simulate daily life activities such as walking and stair climbing. However, it is important to evaluate biomechanical differences within TKA, although exact measurement of knee joint kinematics is not possible in clinical in vivo studies. Furthermore, the sample size of eight may reduce the explanatory power of this experimental setup. However, due to the time and effort of the experimental procedure and the use of human specimens, a sample size of 7–10 is widely used in this research area and so far, has generated several significant findings.

Moreover, a reconstruction of the individual tibial slope within KA with the use of a CR design may contribute to varying amounts of AP stability, as CR TKA is mainly designed for the use with mechanical alignment, targeting a fixed degree of tibial slope (mainly 3°). On the other hand, the use of a mechanical alignment technique may influence the ability of an MP motion pattern with the use of an MP design due to relevant alteration of joint line obliquity and external femoral component rotation. Therefore, our findings can only be transferred to KA TKA.

## CONCLUSION

The MP TKA design contributes to a better reconstruction of a native‐like MP motion pattern by increasing the lateral anterior translation and rotation of the tibia during active knee joint flexion. This is accepted as the native kinematic pattern of the knee joint and thus represents proof of concept for the MP design in KA TKA.

The MP design also reduces quadriceps load, which may imply the functional superiority of the MP TKA to the CR TKA in clinical studies and daily life.

## AUTHOR CONTRIBUTIONS

All authors contributed to the study's conception and design. Johanna‐Maria Simon, Leandra Bauer and Christoph Thorwächter prepared the materials, collected data and analyzed the data. Johanna‐Maria Simon and Leandra Bauer wrote the first draft of the manuscript, and all authors commented on previous versions. All authors read and approved the final manuscript.

## CONFLICT OF INTEREST STATEMENT

Peter E. Müller is a consultant for the Medacta shoulder system and B. Braun Aesculap; this in no way influenced the results of this study. The authors declare no conflicts of interest.

## ETHICS STATEMENT

The study was conducted according to the guidelines of the Declaration of Helsinki. The in vitro study was approved by the Ethics Committee of the University of Munich (#20‐829).

## Supporting information

Supporting information.

## Data Availability

The data that support the findings of this study are available from the corresponding author upon reasonable request.
