# Supplementary material for: Improved quadriceps efficiency with a medial pivot in comparison to a cruciate‐retaining design in total knee arthroplasty
Source: Knee Surg Sports Traumatol Arthrosc. 2025 Feb 13;33(7):2527–36. doi: 10.1002/ksa.12624 (PMC12205411; doi:10.1002/ksa.12624)
Supplement: Supplementary file 1 — Supporting information. [file KSA-33-2527-s001.docx]

**Supplementary Information**

Functional regression analysis for femorotibial kinematics, patellofemoral kinematics and knee joint loading:

| Native Situation | | Cruciate Retaining Design | Medial Pivot Design |
| --- | --- | --- | --- |
| Deviance explained = 28.4 % | 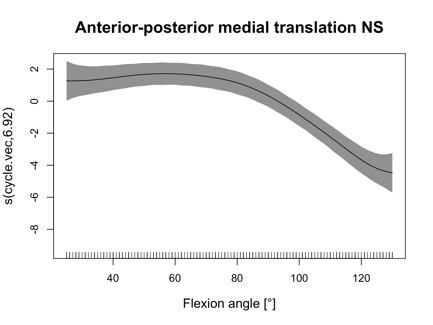  p < 0.001 | 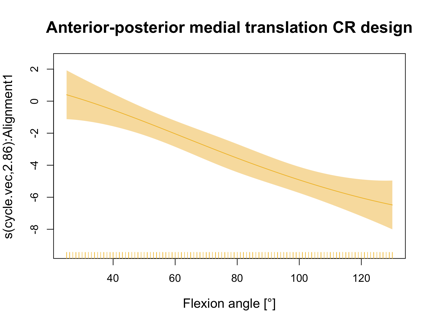  p < 0.001 | 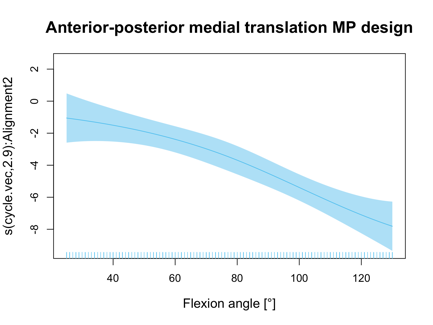  p < 0.001 |
| Deviance explained = 49.5 % | 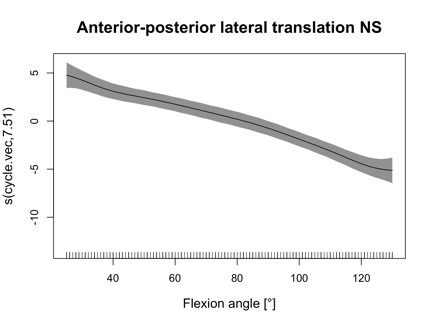  p < 0.001 | 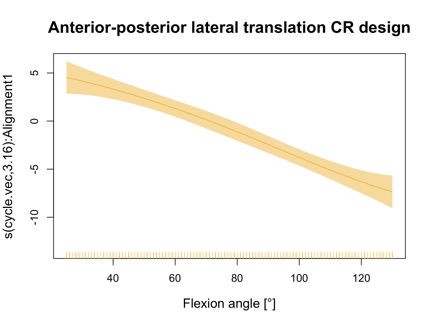p < 0.001 | 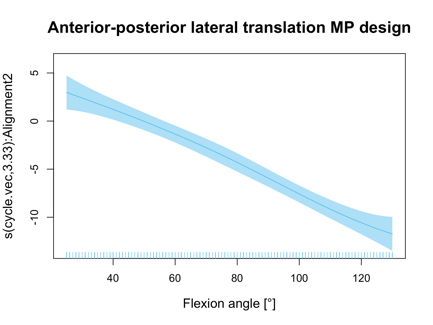p < 0.001 |
| Deviance explained = 43.6 % | 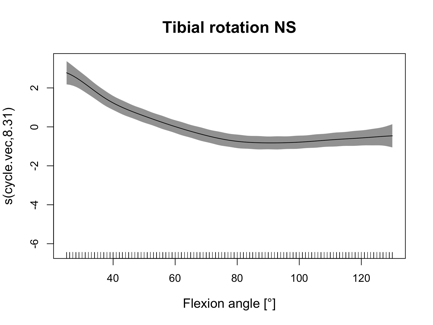  p < 0.001 | 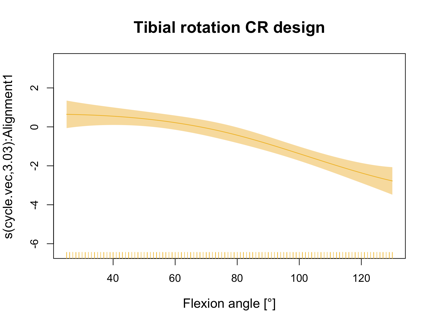p < 0.001 | 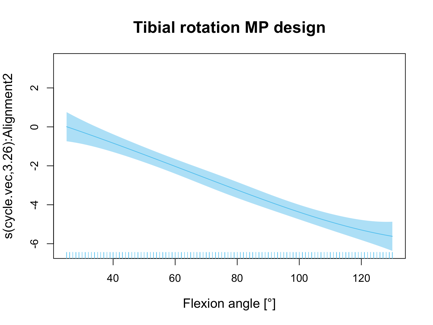p < 0.001 |
| Supplementary Figure 1: Functional regression for femorotibial kinematics – AP medial and lateral translation and tibial rotation – for the intercept (NS), CR design (orange) and MP design (blue). | | | |

| Native Situation | | Cruciate Retaining Design | Medial Pivot Design |
| --- | --- | --- | --- |
| Deviance explained = 17.5 % | 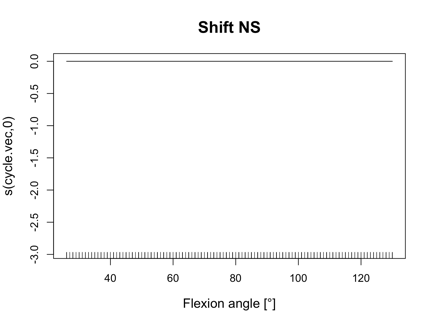  p = 0.68 | 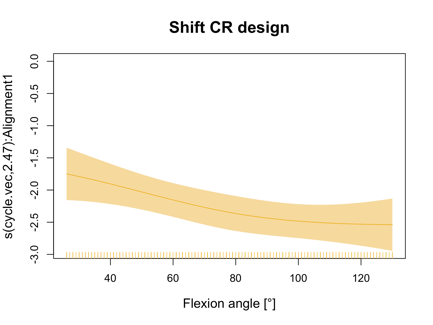  p < 0.001 | 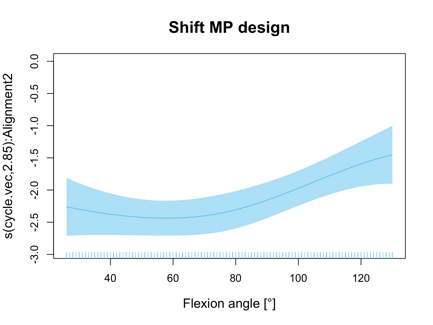  p < 0.001 |
| Deviance explained = 16.1 % | 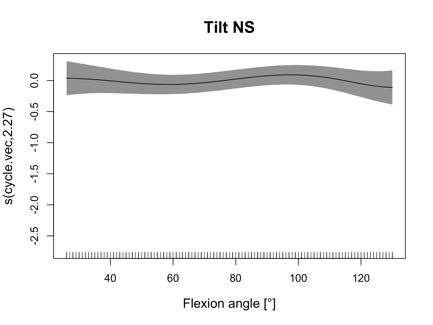  p = 0.12 | 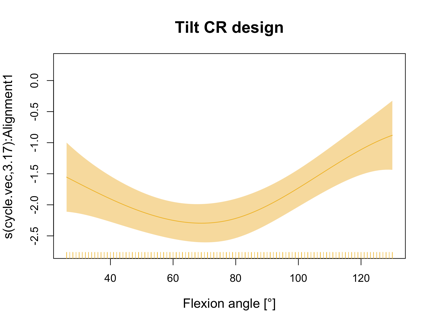  p < 0.001 | 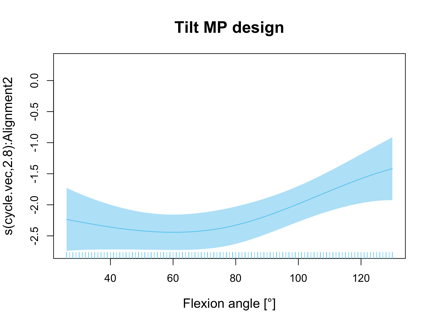  p < 0.001 |
| Supplementary Figure 2: Functional regression for patellofemoral kinematics – shift and tilt – for the intercept (NS), CR design and MP design. | | | |

| Native Situation | | Cruciate Retaining Design | Medial Pivot Design |
| --- | --- | --- | --- |
| Deviance explained = 37.2 % | 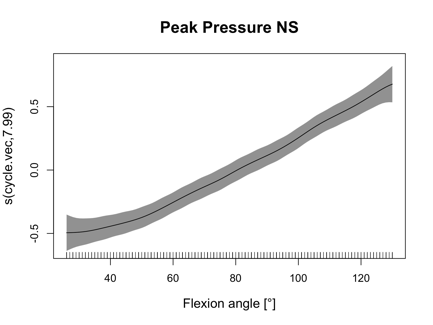  p < 0.001 | 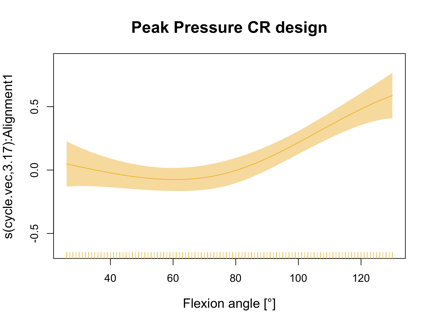  p < 0.001 | 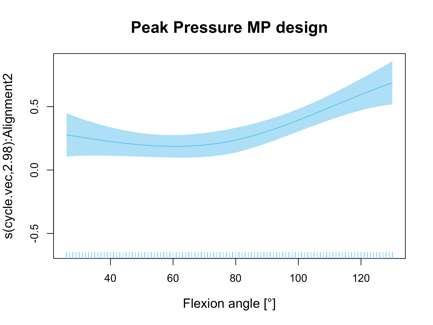  p < 0.001 |
| Deviance explained = 57.3 % | 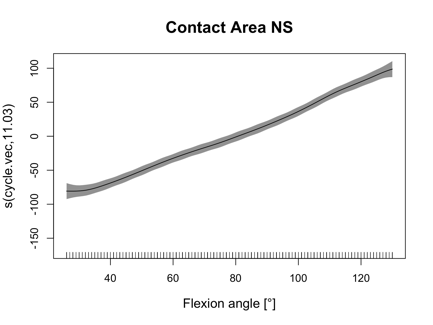  p < 0.001 | 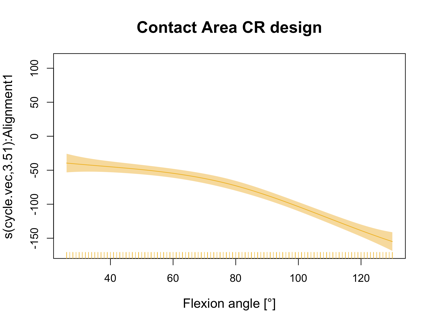  p < 0.001 | 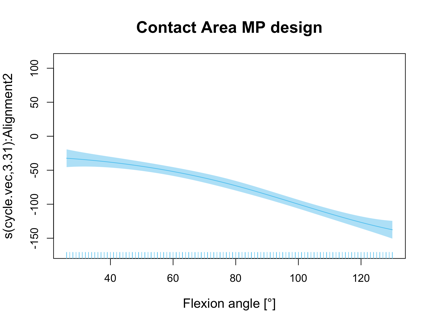p < 0.001 |
| Deviance explained = 89.4 % | 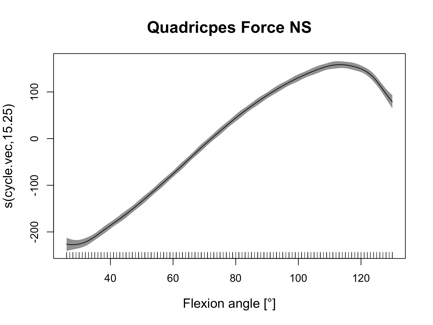  p < 0.001 | 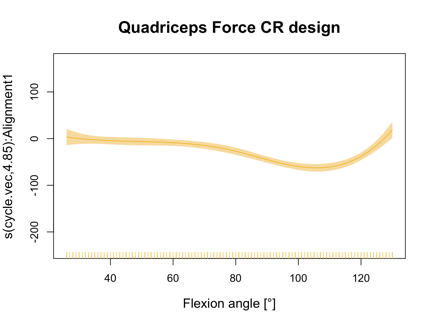  p < 0.001 | 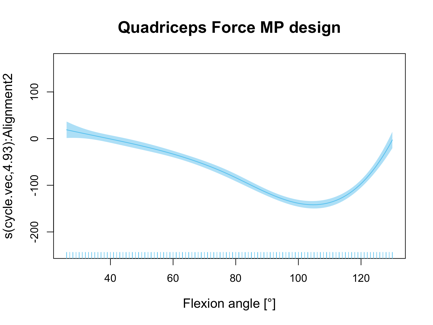  p < 0.001 |
| Supplementary Figure 3: Functional regression for knee joint loading – peak pressure, contact area and quadriceps force – for the intercept (NS), CR design and MP design. | | | |
